# A Rare Presentation of Perivalvular Abscess and Infective Valve Endocarditis as Multiple Cerebral Septic Emboli Mimicking Ischemic Stroke

**DOI:** 10.7759/cureus.41806

**Published:** 2023-07-13

**Authors:** Saikiran Mandyam, Pavan Kumar Reddy Kalluru, Sai Sudha Valisekka, Devam P Parghi

**Affiliations:** 1 Internal Medicine, Southeast Health Medical Center, Dothan, USA; 2 Internal Medicine, Sri Venkateswara Medical College, Tirupati, IND; 3 Internal Medicine, University of Minnesota, Minneapolis, USA

**Keywords:** perivalvular abscess, hemodialysis, end stage renal disease (esrd), catheter related infection, septic emboli, aortic valve infective endocarditis

## Abstract

The perivalvular cardiac abscess is a severe condition associated with infective endocarditis, leading to significant morbidity and mortality if not diagnosed and managed promptly. Neurological complications, particularly stroke, can occur due to embolic events resulting from cardiac abscesses. A 63-year-old female with end-stage renal disease and multiple comorbidities presented with altered mental status. Imaging revealed acute ischemic infarcts in the frontotemporal lobes, suggesting the embolic phenomenon. Blood cultures grew *Enterococcus faecalis*, and an echocardiogram showed severe aortic valve destruction with perivalvular abscess. Cardiac abscesses can cause severe complications, including tissue destruction, valve damage, and embolic events. Echocardiography is crucial for diagnosis, detecting vegetation, and assessing associated complications. Transthoracic echocardiography is reliable but has limitations, whereas transesophageal echocardiography is highly sensitive. Prompt antibiotic therapy and surgical intervention are crucial for treatment. Early initiation of appropriate antibiotic therapy and surgical intervention is crucial for positive outcomes. The choice of treatment should be individualized based on the patient's specific condition and the medical team's expertise.

## Introduction

Conditions such as perivalvular cardiac abscesses are highly morbid and necessitate timely diagnosis and effective management strategies. Infective endocarditis (IE) is widely recognized as the primary cause of cardiac abscesses [1]. Secondary causes of cardiac abscesses include bacteremia (persistent or transient), injured heart tissue following myocardial infarction (MI), and prosthetic valve dysfunction. Less common risk factors include infections at the site of sternal incisions, penetrating wounds, deep burns, trauma, pseudoaneurysms, HIV, and parasite infections [1]. Microbial invasion of the endocardial surface and the formation of abscesses within the cardiac tissue can lead to devastating complications and life-threatening outcomes if left untreated [2]. Prompt suspicion and early intervention are necessary to reduce the mortality rate in patients with intracardiac abscesses. Here, we describe a case with neurological complications due to a perivalvular cardiac abscess in a 63-year-old female.

## Case presentation

**Figure 1:**
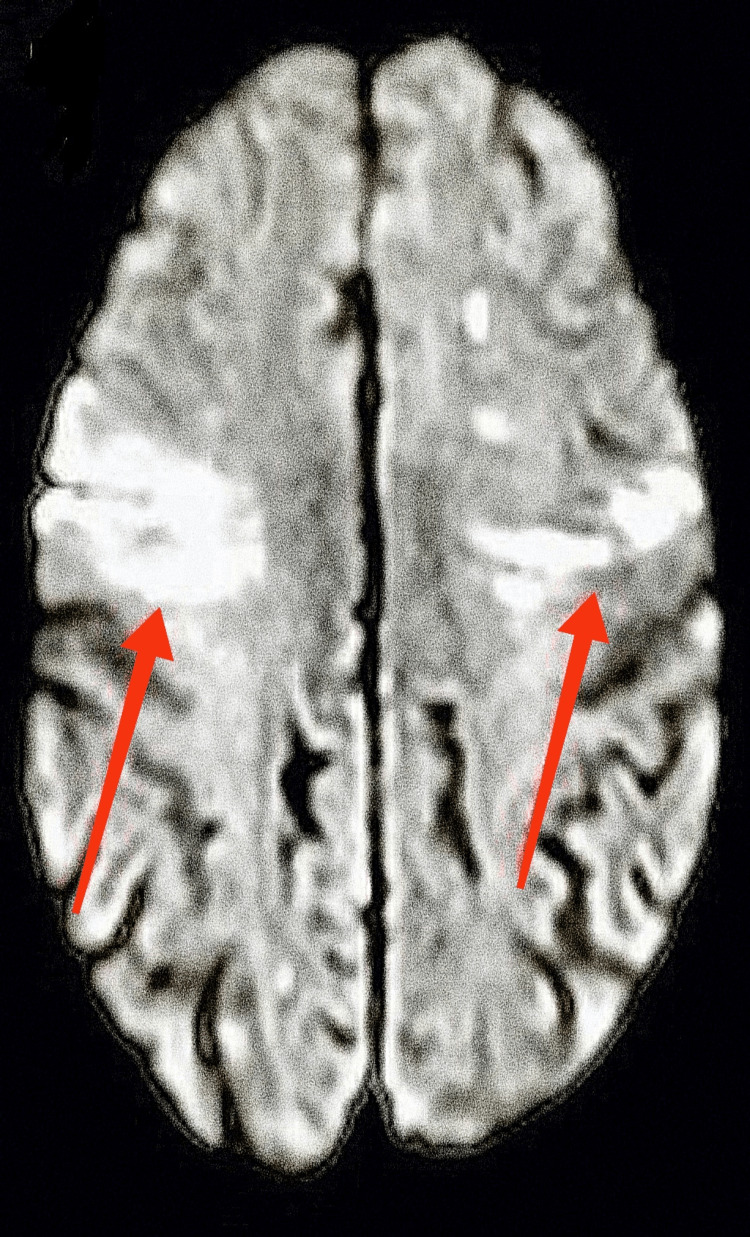
MRI of the head without contrast demonstrating abnormal restriction in the bilateral frontotemporal regions. MRI, magnetic resonance imaging

**Figure 2:**
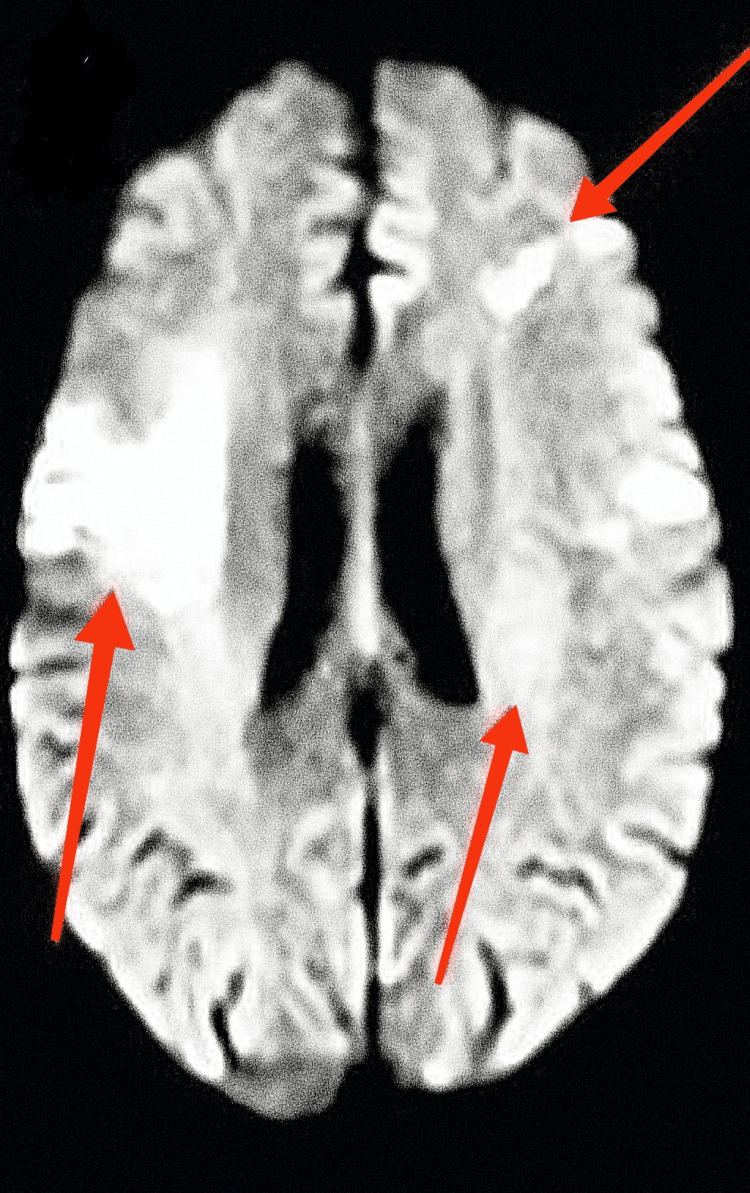
MRI of the head without contrast demonstrating abnormal restriction in the bilateral frontotemporal regions. MRI, magnetic resonance imaging

**Figure 3:**
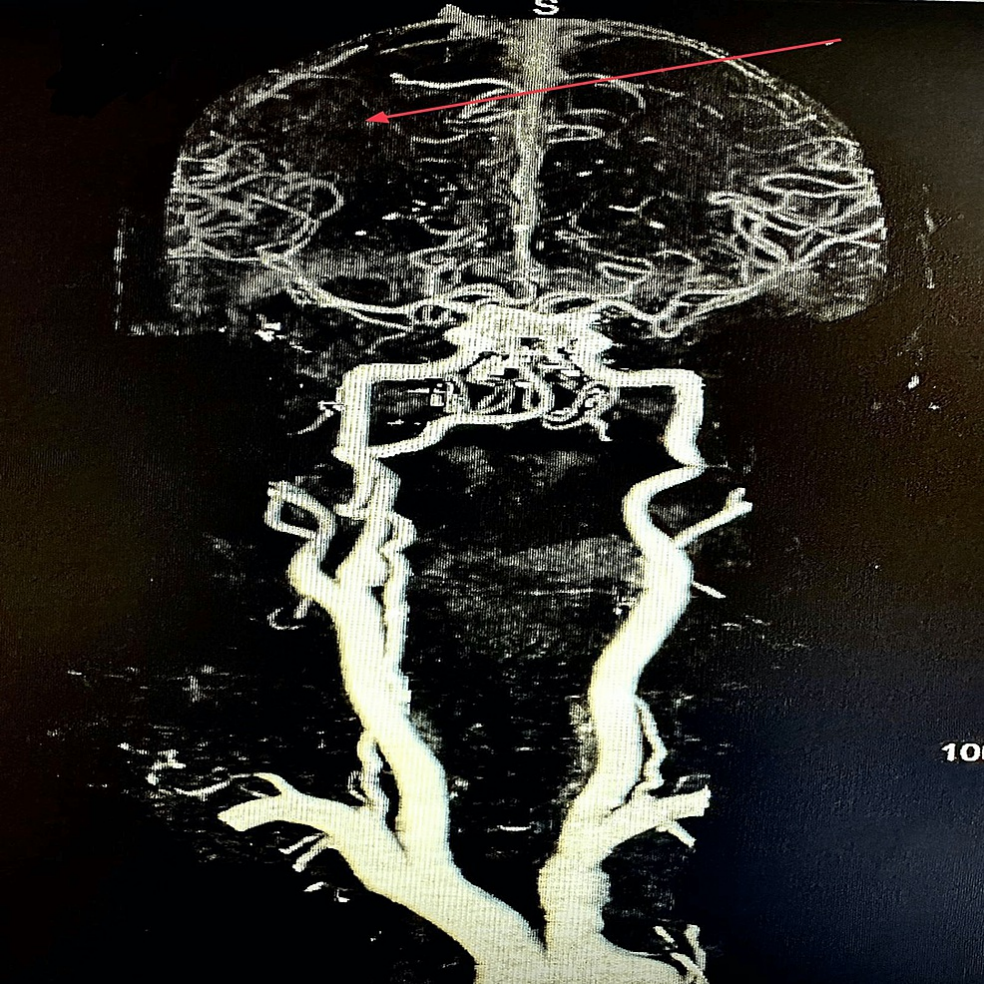
CT angiogram of the head and neck with and without contrast showing decreased right MCA circulation compared to left MCA. CT, computed tomography; MCA, middle cerebral artery

**Figure 4:**
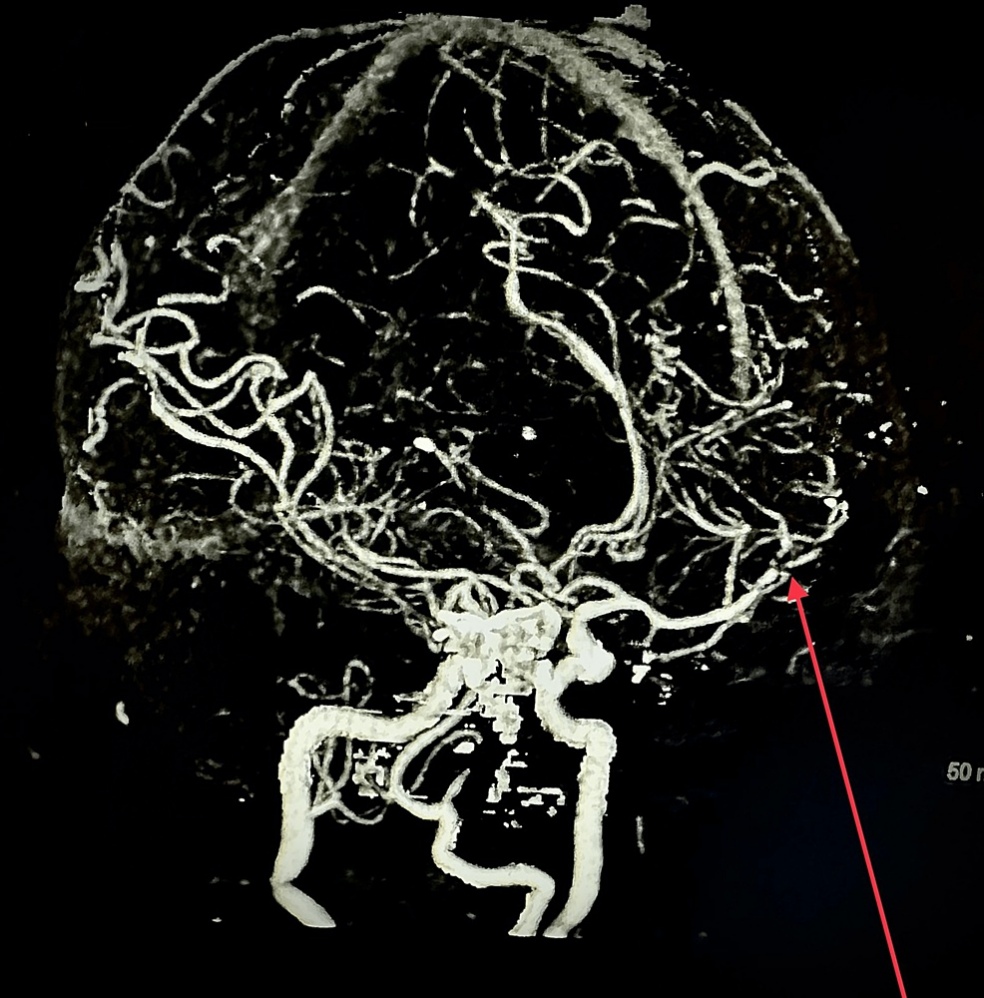
CT angiogram of the head and neck with and without contrast showing high-grade proximal stenosis of the right M3 segment. CT, computed tomography

**Figure 5:**
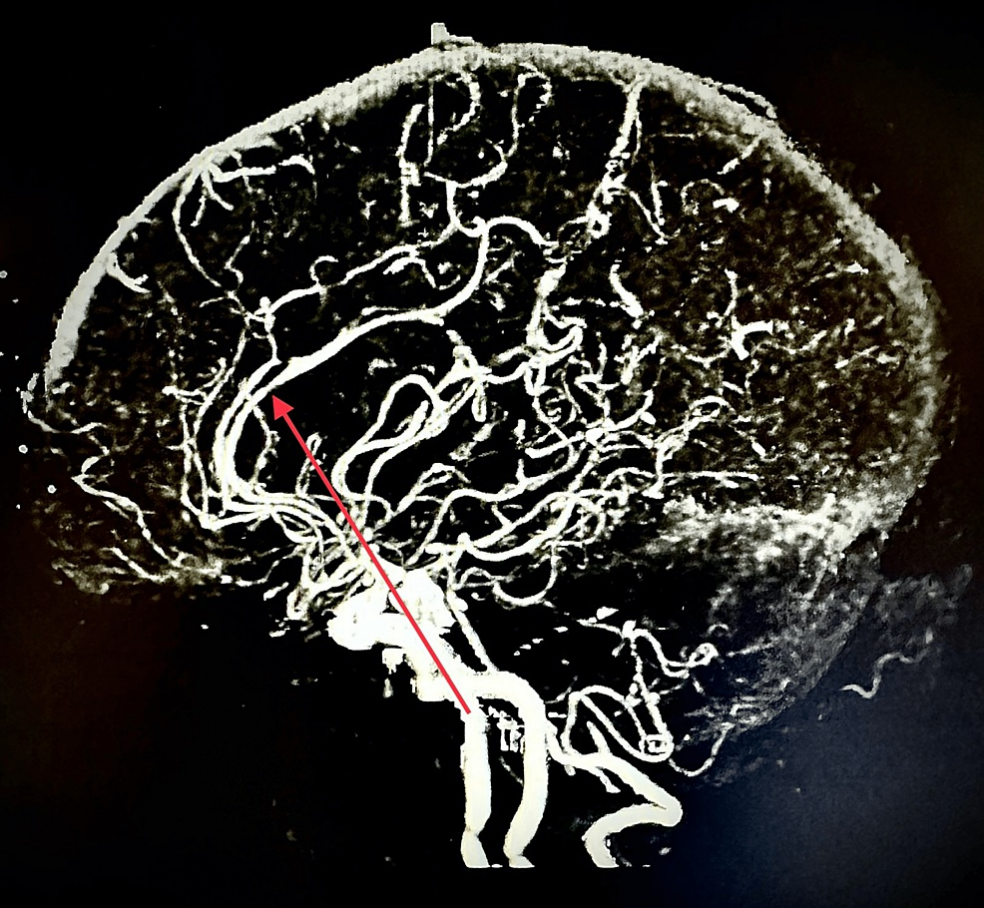
CT angiogram of the head and neck with and without contrast showing focal narrowing (approximately 50%) of the left M2 segment. CT, computed tomography

**Figure 6:**
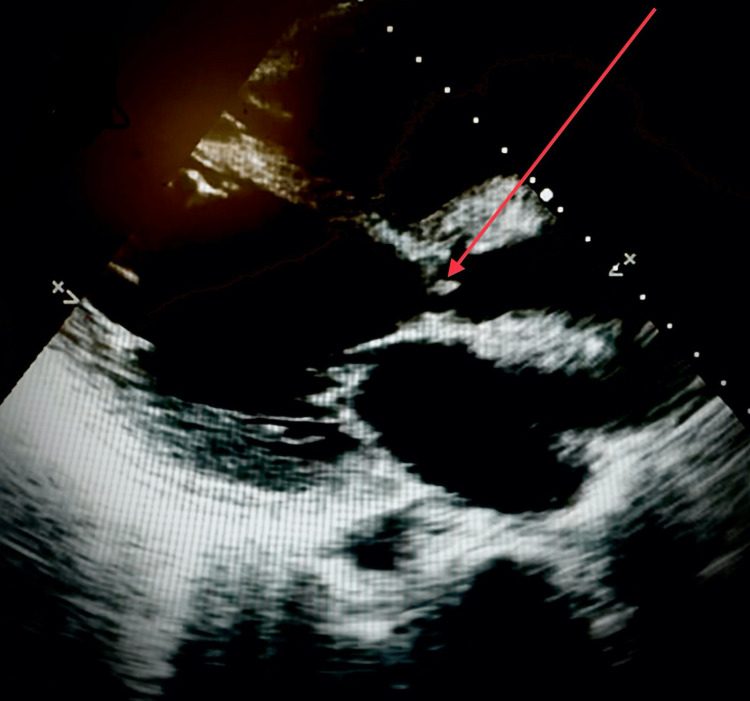
Transthoracic echocardiogram in the parasternal long axis view demonstrating vegetation over the aortic valve with surrounding echolucent area below the origin of the aorta, suspicious for perivalvular abscess.

**Figure 7:**
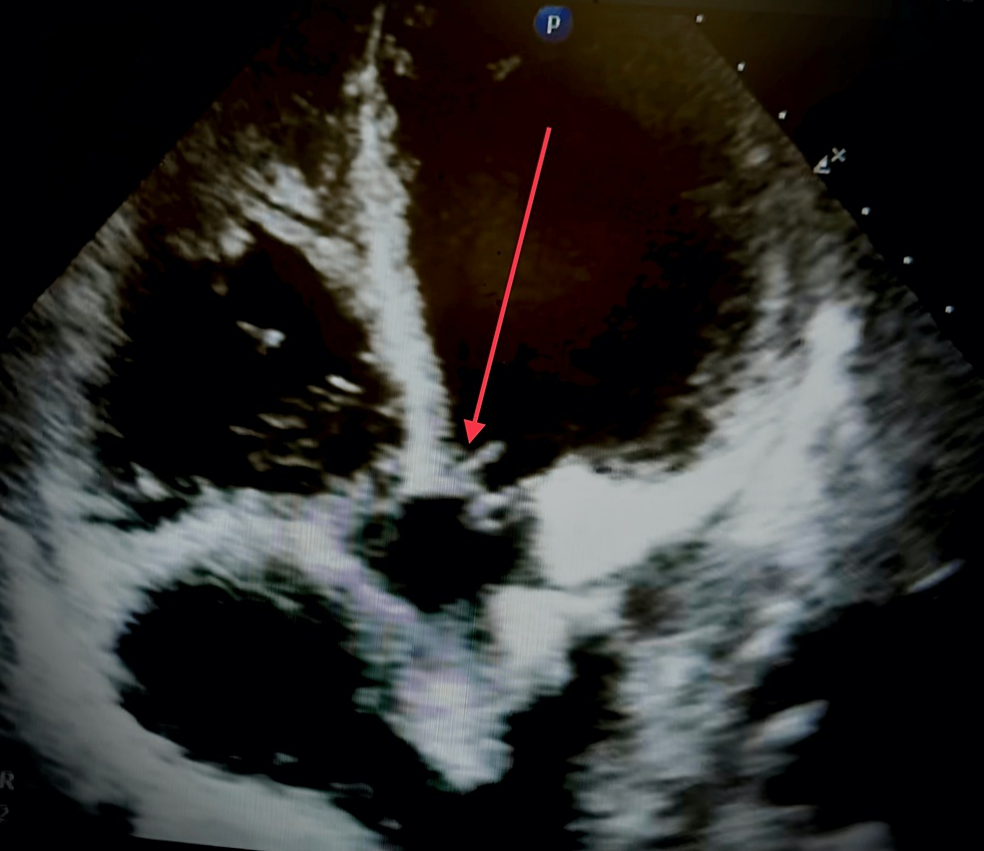
Transthoracic echocardiogram in the four chamber view demonstrating aortic valve vegetation.

A 63-year-old woman with end-stage renal disease, diabetes mellitus type 2, essential hypertension, dyslipidemia, noncompliance with dialysis, and impaired mental status showed up at the office. Forty-eight hours before hospitalization, she was doing good. Computed tomography (CT) of the head was unremarkable for acute abnormality, and magnetic resonance imaging (MRI) of the head without contrast (Figures 1, 2) showed multiple vascular territory acute ischemic infarcts (abnormal restriction predominantly in bilateral frontotemporal lobes) with no hemorrhage, which raised suspicion of the embolic phenomenon. CT angiogram (Figures 3-5) of the head and neck showed normal perfusion in carotid arteries bilaterally, high-grade proximal stenosis of M3 segment, and 50% focal narrowing of the left middle A2 segment. Her white cell count was elevated, and blood cultures grew *Enterococcus faecalis*. Transthoracic echocardiogram (Figures 6, 7) showed aortic valve destruction with surrounding perivalvular abscess resulting in severe aortic regurgitation. Due to the significant risk of bleeding, anticoagulation was not deemed appropriate for her (the patient was on Eliquis 5 mg twice daily at home for unknown reasons). She was started on appropriate antibiotics, with the source of infection presumably being a permanent dialysis catheter. The permanent catheter was removed with plans to insert a temporary dialysis catheter. Unfortunately, the patient's mental status declined, and she became completely unresponsive. Palliative care was involved, and the patient was transitioned to comfort care and passed away with comfort measures.

## Discussion

Cardiac abscesses can lead to significant complications such as destruction of cardiac tissue, valve damage, and formation of vegetation (abnormal growth on valves), resulting in conduction abnormalities, development of new heart murmurs, heart failure, or septic embolic events [3]. Uncommon complications include fistula formation to the pericardial cavity, coronary artery obstruction, and the spread of the abscess to adjacent mediastinal structures [4,5]. Among the complications associated with cardiac abscesses, embolic events are the second most commonly observed after heart failure. The incidence of embolic events ranges from approximately 15% to 35% and can occur one to two years after the abscess resolution [6]. Vegetations on the left side of the heart are usually directed toward the brain and spleen, whereas those on the right side tend to travel to the pulmonary artery [6]. Embolic events targeting the brain can lead to neurological complications, with stroke being the most frequent. Other complications include the formation of small and large abscesses, infectious aneurysms, general toxic-metabolic encephalopathies, increased white blood cells in cerebrospinal fluid (CSF) analysis, and seizures [7]. Neurological complications have implications for diagnosis, management, and prognosis, underscoring the importance of identifying embolic events.

Echocardiography is pivotal in diagnosing cardiac abscess or IE by enabling vegetation detection, assessment of valvular damage, evaluation of resulting hemodynamic abnormalities, and observation of associated complications. Using ultrasound imaging to detect specific vegetation is particularly valuable in guiding management decisions [8]. Statistical analyses indicate that transthoracic echocardiography has a sensitivity of 60% to 75% for vegetation detection, whereas transesophageal echocardiography has a sensitivity exceeding 95% [6]. TTE is a reliable and efficient method for promptly detecting vegetation and providing information regarding its size, number, and location [6]. However, it is essential to acknowledge the limitations of TTE, as the detection rate of vegetation can be influenced by factors such as obesity, chronic obstructive pulmonary disease, chest wall deformities, vegetation characteristics (size and location), presence of prosthetic materials, and the experience and expertise of the examiner in differentiating vegetation [9].

Differentiating vegetation on echocardiography requires distinguishing it from small tumors of heart valve leaflets and calcified lesions in the valve. Cardiac valve calcification is commonly observed in elderly individuals and appears as dense echoes characterized by patches and lumps [6,10]. In contrast, vegetation typically exhibits movement in response to valve opening and closure, with a relatively loose structure and a faint echo. Primary small tumors, such as myxomas and fibroelastosis, are solitary lesions with a regular morphology, often presenting as round shapes. Conversely, vegetation is characterized by irregular shapes and multiple lesions [6,10].

Additionally, vegetation tends to undergo changes during medical treatment, whereas small tumors do not exhibit noticeable modifications [11]. Combining echocardiographic findings with clinical manifestations and disease conditions is essential to diagnose vegetation accurately [6,11]. In addition to echocardiography, delayed contrast-enhanced MRI can offer additional insights such as the spread of infection in antegrade and retrograde directions, expansion of paravalvular tissues, and involvement of the subendocardial and vascular endothelial regions, although it is not commonly employed in routine practice [8]. For the diagnosis of neurologic problems, MRI outperforms brain CT. Recent advances in sensitive MRI sequences can identify cerebral microhemorrhages and microinfarctions [12]. These microhemorrhages are not yet known to have a clear clinical significance. Some MRI neurological abnormalities can be more significant in diagnostic and treatment choices since they are characteristically seen as complications in patients with IE or cardiac abscesses [12].

The prompt beginning of adequate antibiotic therapy is critical in treating intracardiac abscesses. Blood cultures should be obtained to identify the causative microorganisms and guide antibiotic selection, and said blood cultures of this patient revealed Enterococcus faecalis. Until culture results are available, empirical antibiotic therapy should be commenced to cover a broad spectrum of possible infections [13]. However, surgical intervention is also essential to treating intracardiac perivalvular abscesses. If detected early, surgical scheduling has demonstrated that embolic risk decreases considerably during or after the first two to three weeks of effective antibiotic therapy [14]. The surgical method chosen is determined by factors such as the size and location of the abscess. Surgical techniques may include valve repair or replacement and debridement of contaminated tissue [14]. However, studies have reported inpatient mortality rates between 12% and 24% for peri-annular abscesses, irrespective of the surgical technique [15]. Therefore, the choice of antibiotics and surgical intervention should be individualized based on the patient's specific condition and the medical team's expertise. Likewise, patients deemed unfit for surgical procedures should receive individualized management strategies.

## Conclusions

IE and cardiac abscesses are serious and potentially life-threatening conditions that require prompt diagnosis and management. Potentially significant complications including embolic events and neurological complications such as stroke can occur in such patients. Echocardiography is crucial in diagnosing cardiac abscesses by detecting vegetation, assessing vascular damage, and evaluating associated complications. The limitations of echocardiography should be considered, and additional imaging modalities, such as MRI, can provide supplementary information. Prompt initiation of appropriate antibiotic therapy and surgical intervention is crucial in treating intracardiac abscesses. Individualized approaches based on the patient's condition and the medical team's expertise are essential.
